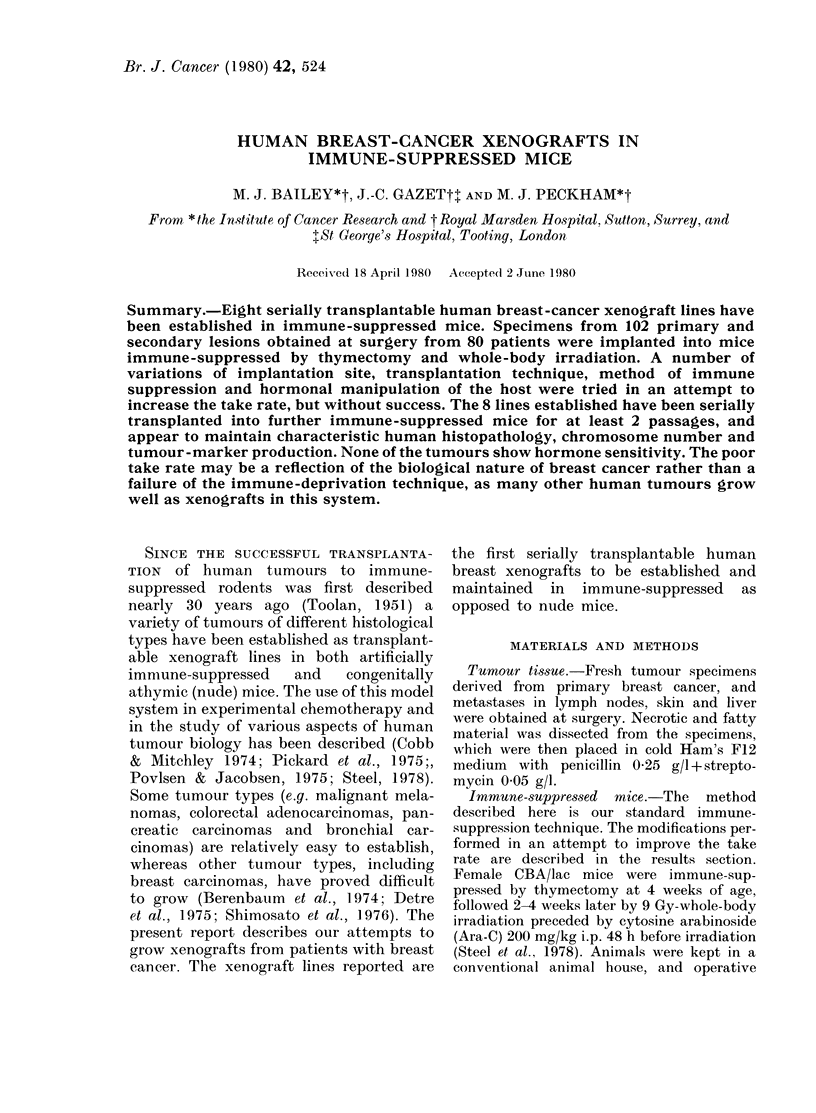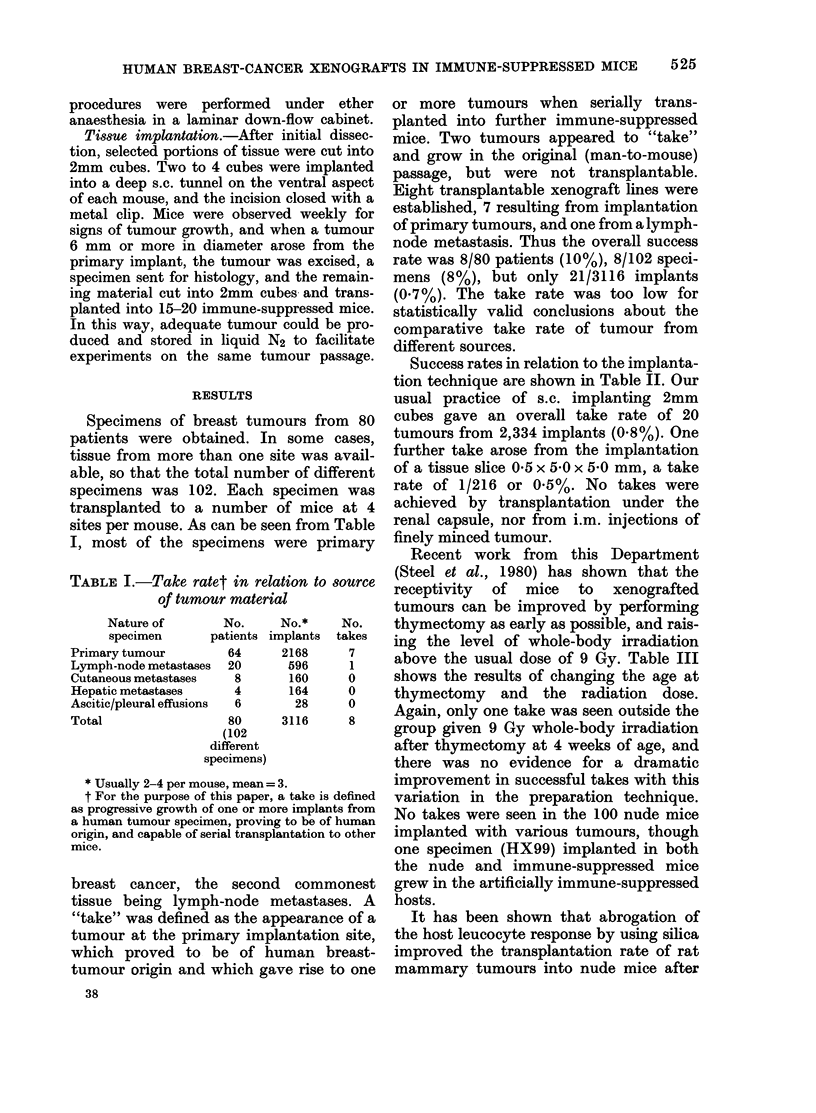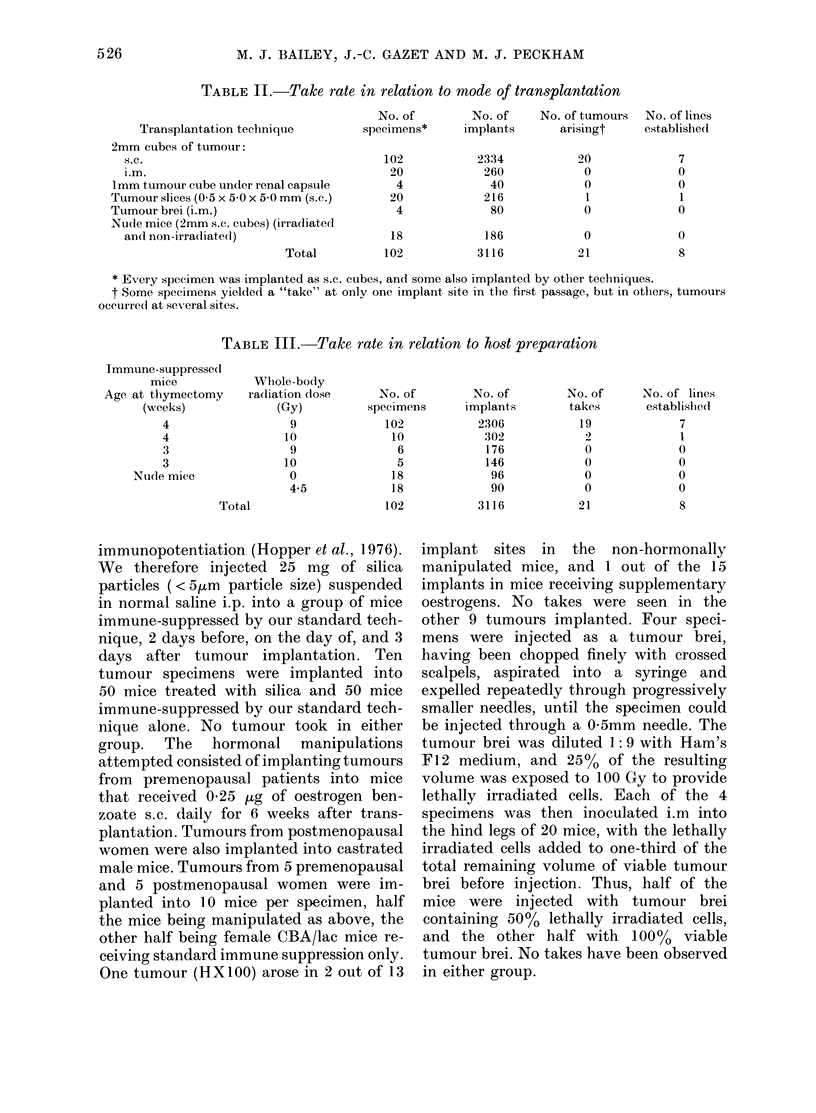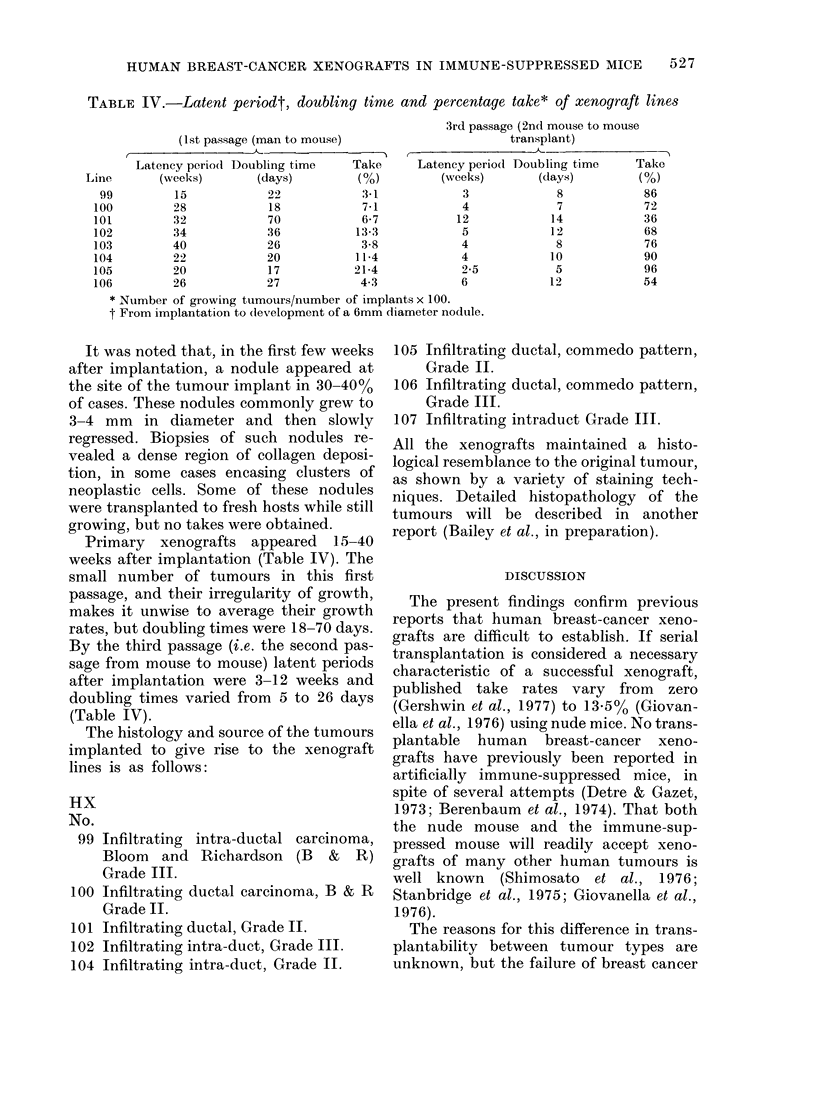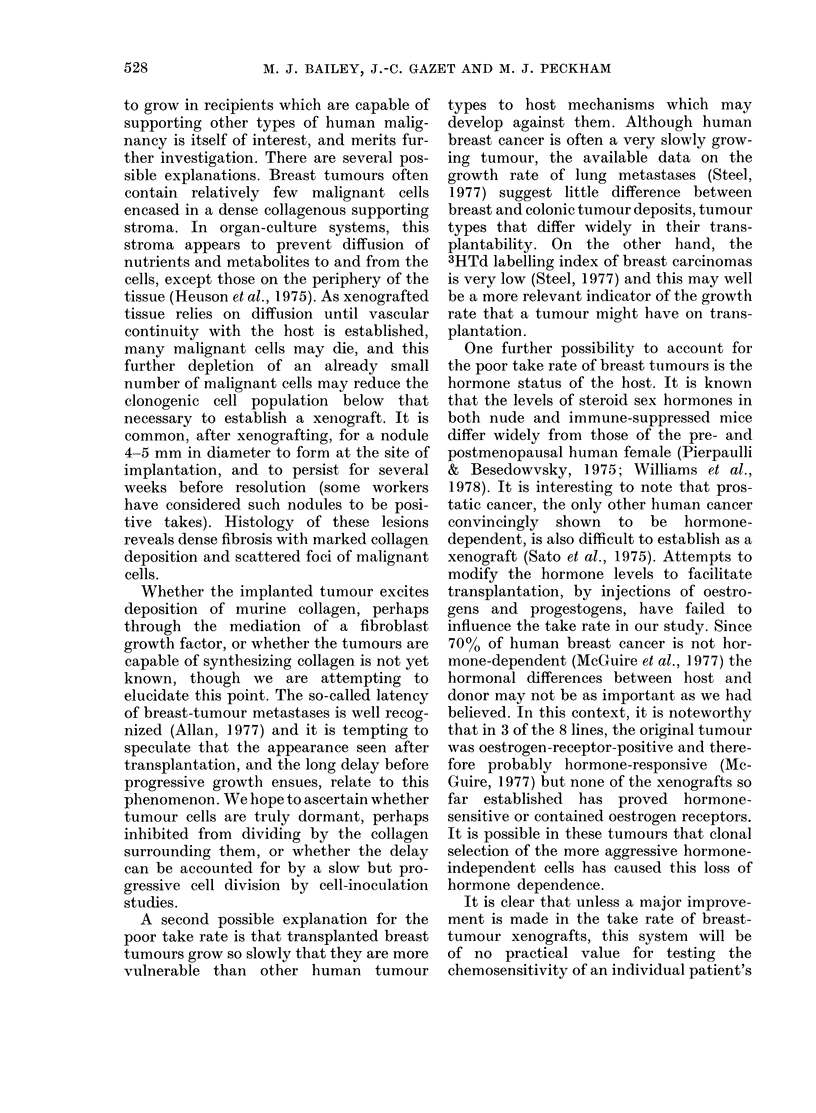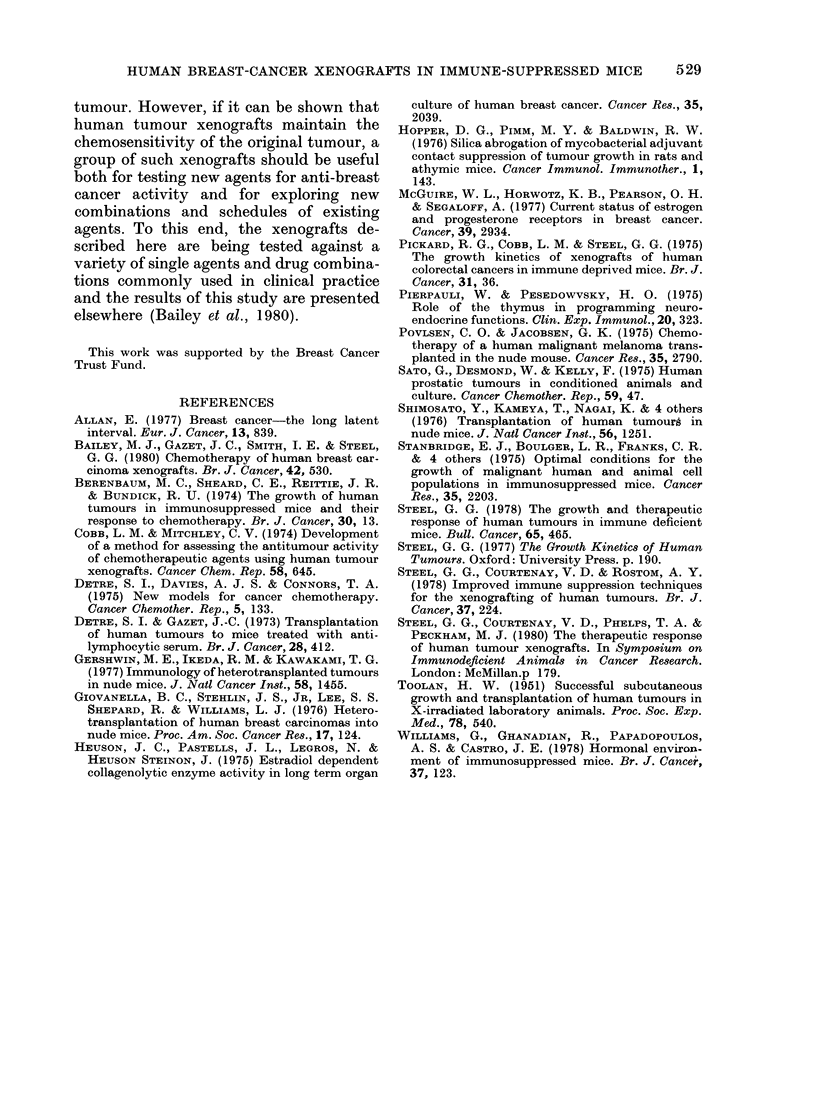# Human breast-cancer xenografts in immune-suppressed mice.

**DOI:** 10.1038/bjc.1980.275

**Published:** 1980-10

**Authors:** M. J. Bailey, J. C. Gazet, M. J. Peckham

## Abstract

Eight serially transplantable human breast-cancer xenograft lines have been established in immune-suppressed mice. Specimens from 102 primary and secondary lesions obtained at surgery from 80 patients were implanted into mice immune-suppressed by thymectomy and whole-body irradiation. A number of variations of implantation site, transplantation technique, method of immune suppression and hormonal manipulation of the host were tried in an attempt to increase the take rate, but without success. The 8 lines established have been serially transplanted into further immune-suppressed mice for at least 2 passages, and appear to maintain characteristic human histopathology, chromosome number and tumour-marker production. None of the tumours show hormone sensitivity. The poor take rate may be a reflection of the biological nature of breast cancer rather than a failure of the immune-deprivation technique, as many other human tumours grow well as xenografts in this system.


*Br. J. Cancer* (1980) **42,** 524

### HUMAN BREAST-CANCER XENOGRAFTS IN IMMUNE-SUPPRESSED MICE

M. J. BAILEY*†, J.-C. GAZET†‡ AND M. J. PECKHAM*†

*From *the Institute of Cancer Research and †Royal Marsden Hospital, Sutton, Surrey, and ‡St George's Hospital, Tooting, London*

Received 18 April 1980 Accepted 2 June 1980

**Summary.—Eight serially transplantable human breast-cancer xenograft lines have been established in immune-suppressed mice. Specimens from 102 primary and secondary lesions obtained at surgery from 80 patients were implanted into mice immune-suppressed by thymectomy and whole-body irradiation. A number of variations of implantation site, transplantation technique, method of immune suppression and hormonal manipulation of the host were tried in an attempt to increase the take rate, but without success. The 8 lines established have been serially transplanted into further immune-suppressed mice for at least 2 passages, and appear to maintain characteristic human histopathology, chromosome number and tumour-marker production. None of the tumours show hormone sensitivity. The poor take rate may be a reflection of the biological nature of breast cancer rather than a failure of the immune-deprivation technique, as many other human tumours grow well as xenografts in this system.**

SINCE THE SUCCESSFUL TRANSPLANTATION of human tumours to immune-suppressed rodents was first described nearly 30 years ago (Toolan, 1951) a variety of tumours of different histological types have been established as transplantable xenograft lines in both artificially immune-suppressed and congenitally athymic (nude) mice. The use of this model system in experimental chemotherapy and in the study of various aspects of human tumour biology has been described (Cobb & Mitchley 1974; Pickard *et al.*, 1975;, Povlsen & Jacobsen, 1975; Steel, 1978). Some tumour types (*e.g.* malignant melanomas, colorectal adenocarcinomas, pancreatic carcinomas and bronchial carcinomas) are relatively easy to establish, whereas other tumour types, including breast carcinomas, have proved difficult to grow (Berenbaum *et al.*, 1974; Detre *et al.*, 1975; Shimosato *et al.*, 1976). The present report describes our attempts to grow xenografts from patients with breast cancer. The xenograft lines reported are the first serially transplantable human breast xenografts to be established and maintained in immune-suppressed as opposed to nude mice.

### MATERIALS AND METHODS

*Tumour tissue.*—Fresh tumour specimens derived from primary breast cancer, and metastases in lymph nodes, skin and liver were obtained at surgery. Necrotic and fatty material was dissected from the specimens, which were then placed in cold Ham's F12 medium with penicillin 0·25 g/l + streptomycin 0·05 g/l.

*Immune-suppressed mice.*—The method described here is our standard immune-suppression technique. The modifications performed in an attempt to improve the take rate are described in the results section. Female CBA/lac mice were immune-suppressed by thymectomy at 4 weeks of age, followed 2–4 weeks later by 9 Gy-whole-body irradiation preceded by cytosine arabinoside (Ara-C) 200 mg/kg i.p. 48 h before irradiation (Steel *et al.*, 1978). Animals were kept in a conventional animal house, and operative procedures were performed under ether anaesthesia in a laminar down-flow cabinet.

*Tissue implantation.*—After initial dissection, selected portions of tissue were cut into 2mm cubes. Two to 4 cubes were implanted into a deep s.c. tunnel on the ventral aspect of each mouse, and the incision closed with a metal clip. Mice were observed weekly for signs of tumour growth, and when a tumour 6 mm or more in diameter arose from the primary implant, the tumour was excised, a specimen sent for histology, and the remaining material cut into 2mm cubes and transplanted into 15–20 immune-suppressed mice. In this way, adequate tumour could be produced and stored in liquid $N_2$ to facilitate experiments on the same tumour passage.

### RESULTS

Specimens of breast tumours from 80 patients were obtained. In some cases, tissue from more than one site was available, so that the total number of different specimens was 102. Each specimen was transplanted to a number of mice at 4 sites per mouse. As can be seen from Table I, most of the specimens were primary

TABLE I.—*Take rate† in relation to source of tumour material*

| Nature of specimen | No. patients | No.* implants | No. takes |
|---|---|---|---|
| Primary tumour | 64 | 2168 | 7 |
| Lymph-node metastases | 20 | 596 | 1 |
| Cutaneous metastases | 8 | 160 | 0 |
| Hepatic metastases | 4 | 164 | 0 |
| Ascitic/pleural effusions | 6 | 28 | 0 |
| Total | 80 (102 different specimens) | 3116 | 8 |

* Usually 2–4 per mouse, mean = 3.

† For the purpose of this paper, a take is defined as progressive growth of one or more implants from a human tumour specimen, proving to be of human origin, and capable of serial transplantation to other mice.

breast cancer, the second commonest tissue being lymph-node metastases. A "take" was defined as the appearance of a tumour at the primary implantation site, which proved to be of human breast-tumour origin and which gave rise to one or more tumours when serially transplanted into further immune-suppressed mice. Two tumours appeared to "take" and grow in the original (man-to-mouse) passage, but were not transplantable. Eight transplantable xenograft lines were established, 7 resulting from implantation of primary tumours, and one from a lymph-node metastasis. Thus the overall success rate was 8/80 patients (10%), 8/102 specimens (8%), but only 21/3116 implants (0·7%). The take rate was too low for statistically valid conclusions about the comparative take rate of tumour from different sources.

Success rates in relation to the implantation technique are shown in Table II. Our usual practice of s.c. implanting 2mm cubes gave an overall take rate of 20 tumours from 2,334 implants (0·8%). One further take arose from the implantation of a tissue slice 0·5 × 5·0 × 5·0 mm, a take rate of 1/216 or 0·5%. No takes were achieved by transplantation under the renal capsule, nor from i.m. injections of finely minced tumour.

Recent work from this Department (Steel *et al.*, 1980) has shown that the receptivity of mice to xenografted tumours can be improved by performing thymectomy as early as possible, and raising the level of whole-body irradiation above the usual dose of 9 Gy. Table III shows the results of changing the age at thymectomy and the radiation dose. Again, only one take was seen outside the group given 9 Gy whole-body irradiation after thymectomy at 4 weeks of age, and there was no evidence for a dramatic improvement in successful takes with this variation in the preparation technique. No takes were seen in the 100 nude mice implanted with various tumours, though one specimen (HX99) implanted in both the nude and immune-suppressed mice grew in the artificially immune-suppressed hosts.

It has been shown that abrogation of the host leucocyte response by using silica improved the transplantation rate of rat mammary tumours into nude mice after

TABLE II.—*Take rate in relation to mode of transplantation*

| Transplantation technique | No. of specimens* | No. of implants | No. of tumours arising† | No. of lines established |
|---|---|---|---|---|
| 2mm cubes of tumour: | | | | |
| s.c. | 102 | 2334 | 20 | 7 |
| i.m. | 20 | 260 | 0 | 0 |
| 1mm tumour cube under renal capsule | 4 | 40 | 0 | 0 |
| Tumour slices (0·5 × 5·0 × 5·0 mm (s.c.) | 20 | 216 | 1 | 1 |
| Tumour brei (i.m.) | 4 | 80 | 0 | 0 |
| Nude mice (2mm s.c. cubes) (irradiated and non-irradiated) | 18 | 186 | 0 | 0 |
| Total | 102 | 3116 | 21 | 8 |

* Every specimen was implanted as s.c. cubes, and some also implanted by other techniques.
† Some specimens yielded a "take" at only one implant site in the first passage, but in others, tumours occurred at several sites.

TABLE III.—*Take rate in relation to host preparation*

| Immune-suppressed mice Age at thymectomy (weeks) | Whole-body radiation dose (Gy) | No. of specimens | No. of implants | No. of takes | No. of lines established |
|---|---|---|---|---|---|
| 4 | 9 | 102 | 2306 | 19 | 7 |
| 4 | 10 | 10 | 302 | 2 | 1 |
| 3 | 9 | 6 | 176 | 0 | 0 |
| 3 | 10 | 5 | 146 | 0 | 0 |
| Nude mice | 0 | 18 | 96 | 0 | 0 |
| | 4·5 | 18 | 90 | 0 | 0 |
| Total | | 102 | 3116 | 21 | 8 |

immunopotentiation (Hopper *et al.*, 1976). We therefore injected 25 mg of silica particles ($< 5 \mu m$ particle size) suspended in normal saline i.p. into a group of mice immune-suppressed by our standard technique, 2 days before, on the day of, and 3 days after tumour implantation. Ten tumour specimens were implanted into 50 mice treated with silica and 50 mice immune-suppressed by our standard technique alone. No tumour took in either group. The hormonal manipulations attempted consisted of implanting tumours from premenopausal patients into mice that received 0·25 $\mu$g of oestrogen benzoate s.c. daily for 6 weeks after transplantation. Tumours from postmenopausal women were also implanted into castrated male mice. Tumours from 5 premenopausal and 5 postmenopausal women were implanted into 10 mice per specimen, half the mice being manipulated as above, the other half being female CBA/lac mice receiving standard immune suppression only. One tumour (HX100) arose in 2 out of 13 implant sites in the non-hormonally manipulated mice, and 1 out of the 15 implants in mice receiving supplementary oestrogens. No takes were seen in the other 9 tumours implanted. Four specimens were injected as a tumour brei, having been chopped finely with crossed scalpels, aspirated into a syringe and expelled repeatedly through progressively smaller needles, until the specimen could be injected through a 0·5mm needle. The tumour brei was diluted 1 : 9 with Ham's F12 medium, and 25% of the resulting volume was exposed to 100 Gy to provide lethally irradiated cells. Each of the 4 specimens was then inoculated i.m into the hind legs of 20 mice, with the lethally irradiated cells added to one-third of the total remaining volume of viable tumour brei before injection. Thus, half of the mice were injected with tumour brei containing 50% lethally irradiated cells, and the other half with 100% viable tumour brei. No takes have been observed in either group.

TABLE IV.—*Latent period*†, *doubling time and percentage take* of xenograft lines*

| Line | (1st passage (man to mouse) Latency period (weeks) | Doubling time (days) | Take (%) | 3rd passage (2nd mouse to mouse transplant) Latency period (weeks) | Doubling time (days) | Take (%) |
|---|---|---|---|---|---|---|
| 99 | 15 | 22 | 3·1 | 3 | 8 | 86 |
| 100 | 28 | 18 | 7·1 | 4 | 7 | 72 |
| 101 | 32 | 70 | 6·7 | 12 | 14 | 36 |
| 102 | 34 | 36 | 13·3 | 5 | 12 | 68 |
| 103 | 40 | 26 | 3·8 | 4 | 8 | 76 |
| 104 | 22 | 20 | 11·4 | 4 | 10 | 90 |
| 105 | 20 | 17 | 21·4 | 2·5 | 5 | 96 |
| 106 | 26 | 27 | 4·3 | 6 | 12 | 54 |

* Number of growing tumours/number of implants × 100.
† From implantation to development of a 6mm diameter nodule.

It was noted that, in the first few weeks after implantation, a nodule appeared at the site of the tumour implant in 30–40% of cases. These nodules commonly grew to 3–4 mm in diameter and then slowly regressed. Biopsies of such nodules revealed a dense region of collagen deposition, in some cases encasing clusters of neoplastic cells. Some of these nodules were transplanted to fresh hosts while still growing, but no takes were obtained.

Primary xenografts appeared 15–40 weeks after implantation (Table IV). The small number of tumours in this first passage, and their irregularity of growth, makes it unwise to average their growth rates, but doubling times were 18–70 days. By the third passage (*i.e.* the second passage from mouse to mouse) latent periods after implantation were 3–12 weeks and doubling times varied from 5 to 26 days (Table IV).

The histology and source of the tumours implanted to give rise to the xenograft lines is as follows:

HX No.

99 Infiltrating intra-ductal carcinoma, Bloom and Richardson (B & R) Grade III.
100 Infiltrating ductal carcinoma, B & R Grade II.
101 Infiltrating ductal, Grade II.
102 Infiltrating intra-duct, Grade III.
104 Infiltrating intra-duct, Grade II.
105 Infiltrating ductal, commedo pattern, Grade II.
106 Infiltrating ductal, commedo pattern, Grade III.
107 Infiltrating intraduct Grade III.

All the xenografts maintained a histological resemblance to the original tumour, as shown by a variety of staining techniques. Detailed histopathology of the tumours will be described in another report (Bailey *et al.*, in preparation).

### DISCUSSION

The present findings confirm previous reports that human breast-cancer xenografts are difficult to establish. If serial transplantation is considered a necessary characteristic of a successful xenograft, published take rates vary from zero (Gershwin *et al.*, 1977) to 13·5% (Giovanella *et al.*, 1976) using nude mice. No transplantable human breast-cancer xenografts have previously been reported in artificially immune-suppressed mice, in spite of several attempts (Detre & Gazet, 1973; Berenbaum *et al.*, 1974). That both the nude mouse and the immune-suppressed mouse will readily accept xenografts of many other human tumours is well known (Shimosato *et al.*, 1976; Stanbridge *et al.*, 1975; Giovanella *et al.*, 1976).

The reasons for this difference in transplantability between tumour types are unknown, but the failure of breast cancer to grow in recipients which are capable of supporting other types of human malignancy is itself of interest, and merits further investigation. There are several possible explanations. Breast tumours often contain relatively few malignant cells encased in a dense collagenous supporting stroma. In organ-culture systems, this stroma appears to prevent diffusion of nutrients and metabolites to and from the cells, except those on the periphery of the tissue (Heuson *et al.*, 1975). As xenografted tissue relies on diffusion until vascular continuity with the host is established, many malignant cells may die, and this further depletion of an already small number of malignant cells may reduce the clonogenic cell population below that necessary to establish a xenograft. It is common, after xenografting, for a nodule 4–5 mm in diameter to form at the site of implantation, and to persist for several weeks before resolution (some workers have considered such nodules to be positive takes). Histology of these lesions reveals dense fibrosis with marked collagen deposition and scattered foci of malignant cells.

Whether the implanted tumour excites deposition of murine collagen, perhaps through the mediation of a fibroblast growth factor, or whether the tumours are capable of synthesizing collagen is not yet known, though we are attempting to elucidate this point. The so-called latency of breast-tumour metastases is well recognized (Allan, 1977) and it is tempting to speculate that the appearance seen after transplantation, and the long delay before progressive growth ensues, relate to this phenomenon. We hope to ascertain whether tumour cells are truly dormant, perhaps inhibited from dividing by the collagen surrounding them, or whether the delay can be accounted for by a slow but progressive cell division by cell-inoculation studies.

A second possible explanation for the poor take rate is that transplanted breast tumours grow so slowly that they are more vulnerable than other human tumour types to host mechanisms which may develop against them. Although human breast cancer is often a very slowly growing tumour, the available data on the growth rate of lung metastases (Steel, 1977) suggest little difference between breast and colonic tumour deposits, tumour types that differ widely in their transplantability. On the other hand, the $^{3}$HTd labelling index of breast carcinomas is very low (Steel, 1977) and this may well be a more relevant indicator of the growth rate that a tumour might have on transplantation.

One further possibility to account for the poor take rate of breast tumours is the hormone status of the host. It is known that the levels of steroid sex hormones in both nude and immune-suppressed mice differ widely from those of the pre- and postmenopausal human female (Pierpaulli & Besedowvsky, 1975; Williams *et al.*, 1978). It is interesting to note that prostatic cancer, the only other human cancer convincingly shown to be hormone-dependent, is also difficult to establish as a xenograft (Sato *et al.*, 1975). Attempts to modify the hormone levels to facilitate transplantation, by injections of oestrogens and progestogens, have failed to influence the take rate in our study. Since 70% of human breast cancer is not hormone-dependent (McGuire *et al.*, 1977) the hormonal differences between host and donor may not be as important as we had believed. In this context, it is noteworthy that in 3 of the 8 lines, the original tumour was oestrogen-receptor-positive and therefore probably hormone-responsive (McGuire, 1977) but none of the xenografts so far established has proved hormone-sensitive or contained oestrogen receptors. It is possible in these tumours that clonal selection of the more aggressive hormone-independent cells has caused this loss of hormone dependence.

It is clear that unless a major improvement is made in the take rate of breast-tumour xenografts, this system will be of no practical value for testing the chemosensitivity of an individual patient's tumour. However, if it can be shown that human tumour xenografts maintain the chemosensitivity of the original tumour, a group of such xenografts should be useful both for testing new agents for anti-breast cancer activity and for exploring new combinations and schedules of existing agents. To this end, the xenografts described here are being tested against a variety of single agents and drug combinations commonly used in clinical practice and the results of this study are presented elsewhere (Bailey *et al.*, 1980).

This work was supported by the Breast Cancer Trust Fund.

### REFERENCES

ALLAN, E. (1977) Breast cancer—the long latent interval. *Eur. J. Cancer*, **13**, 839.

BAILEY, M. J., GAZET, J. C., SMITH, I. E. & STEEL, G. G. (1980) Chemotherapy of human breast carcinoma xenografts. *Br. J. Cancer*, **42**, 530.

BERENBAUM, M. C., SHEARD, C. E., REITTIE, J. R. & BUNDICK, R. U. (1974) The growth of human tumours in immunosuppressed mice and their response to chemotherapy. *Br. J. Cancer*, **30**, 13.

COBB, L. M. & MITCHLEY, C. V. (1974) Development of a method for assessing the antitumour activity of chemotherapeutic agents using human tumour xenografts. *Cancer Chem. Rep.* **58**, 645.

DETRE, S. I., DAVIES, A. J. S. & CONNORS, T. A. (1975) New models for cancer chemotherapy. *Cancer Chemother. Rep.*, **5**, 133.

DETRE, S. I. & GAZET, J.-C. (1973) Transplantation of human tumours to mice treated with antilymphocytic serum. *Br. J. Cancer*, **28**, 412.

GERSHWIN, M. E., IKEDA, R. M. & KAWAKAMI, T. G. (1977) Immunology of heterotransplanted tumours in nude mice. *J. Natl Cancer Inst.*, **58**, 1455.

GIOVANELLA, B. C., STEHLIN, J. S., JR, LEE, S. S. SHEPARD, R. & WILLIAMS, L. J. (1976) Heterotransplantation of human breast carcinomas into nude mice. *Proc. Am. Soc. Cancer Res.*, **17**, 124.

HEUSON, J. C., PASTELLS, J. L., LEGROS, N. & HEUSON STEINON, J. (1975) Estradiol dependent collagenolytic enzyme activity in long term organ culture of human breast cancer. *Cancer Res.*, **35**, 2039.

HOPPER, D. G., PIMM, M. Y. & BALDWIN, R. W. (1976) Silica abrogation of mycobacterial adjuvant contact suppression of tumour growth in rats and athymic mice. *Cancer Immunol. Immunother.*, **1**, 143.

McGUIRE, W. L., HORWOTZ, K. B., PEARSON, O. H. & SEGALOFF, A. (1977) Current status of estrogen and progesterone receptors in breast cancer. *Cancer*, **39**, 2934.

PICKARD, R. G., COBB, L. M. & STEEL, G. G. (1975) The growth kinetics of xenografts of human colorectal cancers in immune deprived mice. *Br. J. Cancer*, **31**, 36.

PIERPAULI, W. & PESEDOWVSKY, H. O. (1975) Role of the thymus in programming neuroendocrine functions. *Clin. Exp. Immunol.*, **20**, 323.

POVLSEN, C. O. & JACOBSEN, G. K. (1975) Chemotherapy of a human malignant melanoma transplanted in the nude mouse. *Cancer Res.*, **35**, 2790.

SATO, G., DESMOND, W. & KELLY, F. (1975) Human prostatic tumours in conditioned animals and culture. *Cancer Chemother. Rep.*, **59**, 47.

SHIMOSATO, Y., KAMEYA, T., NAGAI, K. & 4 others (1976) Transplantation of human tumours in nude mice. *J. Natl Cancer Inst.*, **56**, 1251.

STANBRIDGE, E. J., BOULGER, L. R., FRANKS, C. R. & 4 others (1975) Optimal conditions for the growth of malignant human and animal cell populations in immunosuppressed mice. *Cancer Res.*, **35**, 2203.

STEEL, G. G. (1978) The growth and therapeutic response of human tumours in immune deficient mice. *Bull. Cancer*, **65**, 465.

STEEL, G. G. (1977) *The Growth Kinetics of Human Tumours*. Oxford: University Press. p. 190.

STEEL, G. G., COURTENAY, V. D. & ROSTOM, A. Y. (1978) Improved immune suppression techniques for the xenografting of human tumours. *Br. J. Cancer*, **37**, 224.

STEEL, G. G., COURTENAY, V. D., PHELPS, T. A. & PECKHAM, M. J. (1980) The therapeutic response of human tumour xenografts. In *Symposium on Immunodeficient Animals in Cancer Research*. London: McMillan.p 179.

TOOLAN, H. W. (1951) Successful subcutaneous growth and transplantation of human tumours in X-irradiated laboratory animals. *Proc. Soc. Exp. Med.*, **78**, 540.

WILLIAMS, G., GHANADIAN, R., PAPADOPOULOS, A. S. & CASTRO, J. E. (1978) Hormonal environment of immunosuppressed mice. *Br. J. Cancer*, **37**, 123.